# Pharmacy Practice and Education in the Czech Republic †

**DOI:** 10.3390/pharmacy5040054

**Published:** 2017-10-09

**Authors:** Petr Nachtigal, Tomáš Šimůnek, Jeffrey Atkinson

**Affiliations:** 1 Faculty of Pharmacy, Charles University, Akademika Heyrovského 1203, Hradec Králové 500 05, Czech Republic; Petr.Nachtigal@faf.cuni.cz (P.N.); Tomas.Simunek@faf.cuni.cz (T.Š.); 2 Pharmacolor Consultants Nancy, 12 rue de Versigny, 54600 Villers, France

**Keywords:** pharmacy, education, practice, Czech Republic

## Abstract

The PHARMINE (“Pharmacy Education in Europe”) project studied the organisation of pharmacy education, practice and legislation in the European Union (EU) with the objectives of evaluating to what degree harmonisation had taken place with the EU, and producing documents on each individual EU member state. Part of this work was in the form of a survey of pharmacy education, practice, and legislation in the various member states. We will publish the individual member state surveys as reference documents. This paper presents the results of the PHARMINE survey on pharmacy education, training, and practice in the Czech Republic. Czech community pharmacies sell and provide advice on Rx and Over-the-counter (OTC) medicines; they also provide diagnostic services (e.g., blood pressure measurement). Pharmacists (*lékárník* in Czech) study for five years and graduate with a *Magister* (Mgr., equivalent to M.Pharm.) degree. The Mgr. diploma is the only requirement for registration as a pharmacist. Pharmacists can own and manage community pharmacies, or work as responsible pharmacists in pharmacies. All practising pharmacists must be registered with the Czech Chamber of Pharmacists. The ownership of a community pharmacy is not restricted to members of the pharmacy profession; the majority of pharmacies are organised into various pharmacy chains. There are two universities providing higher education in pharmacy in the Czech Republic: the Faculty of Pharmacy in Hradec Kralove, Charles University, which was established in 1969, and the Faculty of Pharmacy of the University of Veterinary and Pharmaceutical Sciences in Brno, which was established in 1991. The pharmacy curriculum is organized as a seamless, fully integrated, five-year master degree course. There is a six-month traineeship supervised by the university, which usually takes place during the fifth year. Thus, the pharmacy curriculum is organised in accordance with the EU directive on sectoral professions that lays down the imperatives for pharmacy education, training, and practice in the various member states of the EU. Currently, no specialisation courses are available at the university level. Specialisation is organised in the form of postgraduate, continuing professional development by the Czech Chamber of Pharmacists, and delivered by the Institute of Postgraduate Education for Health Professions.

## 1. Introduction

The PHARMINE (“Pharmacy Education in Europe”) consortium surveyed the state of pharmacy education and practice in the member states of the EU, including the Czech Republic, in 2012, with an update in 2017. The methodology used in the PHARMINE study and the principal results obtained have already been published [1].

The PHARMINE consortium was interested in general practice and education, and in specialisation in pharmacy education for hospital and industrial pharmacy practice. Pharmacy education, training, and practice in the EU are unique in that they fall under two jurisdictions. As for other sectoral professions such as medicine, the European Commission issues directives on the education and training for the sectoral profession of pharmacy [2]. This directive lays down the broad imperatives for education and practice in the EU. An EU directive is a legal act that requires member states to achieve a particular result (in this case, the harmonisation of pharmacy training and practice) without dictating the means of achieving that result. Directives leave the 28 member states with an amount of leeway as to the exact laws and rules to be adopted. Member states may also embrace legislation on specific national practices, such as those relating to specialisation, as well as the ownership and management of community pharmacies.

The situation in Europe is further developed by the Bologna agreement on the harmonisation of the various European degree courses, and student and staff exchange [3]. The Bologna agreement, signed by the education ministers of the governments of the 48 members of the European Higher Education Area (which includes the 28 EU member states), proposes a bachelor (three years) plus master (two years) degree structure for all of the degrees, including pharmacy. This agreement is in opposition to the EU directive that stipulates a five-year “tunnel” degree structure for pharmacy, i.e., a degree course that has no possibility for intermediate entry or exit, for example, after a three-year bachelor period. Another aspect of the Bologna process is the development of tools to promote exchange, such as the European Credit Transfer and Accumulation System (ECTS), which provides credits to students for defined learning outcomes and their associated workload. Another tool is the Diploma Supplement, which provides a description of the nature, level, context, content, and status of the studies that were successfully completed by the student. These systems allow students to study for several months in another university in another EU member state and—importantly—to validate such studies carried out in their host university by their home university. This paper looks at how this system has developed in Czech universities.

The PHARMINE report also dealt with other personnel working in pharmacies, such as assistant pharmacists; their education, training, and responsibilities were surveyed.

In the light of the context described above, it is particularly interesting to examine how this affects pharmacy education and practice in a country—in this instance, the Czech Republic—that recently joined (in 2004) the EU.

Regarding the general health situation in the Czech Republic compared to the EU, life expectancy at birth (see Table 1) in the Czech Republic is only slightly lower than the EU average of 79.4 years, as is healthy life expectancy (EU average 70.2 years). However, it is worth noting that the expenditure on health is 33% lower than the EU average ($3611 per capita). As seen above, health expenditure is mainly in the public sector ($2013) rather than in the private sector ($69).

## 2. Design

Information was obtained from various sources who replied to a questionnaire on pharmacy practice (community, hospital, and industrial), pharmacy organisation and legislation, pharmacy education and training, and finally the impact of the adoption of the Bologna declaration and the EU directive on the sectoral practice of pharmacy. The latter included information on the organisation of the degree course with or without the existence of a bachelor/master structure, and also on the effect of ECTS and the Erasmus programme on student and staff exchange [6].

The information is presented in the form of tables in order to facilitate legibility. This form of presentation was developed in association with this journal’s editorial direction, and has been described in detail in a previous publication [7]. This presentation will ease: the consultation of these country profiles by students and staff envisaging exchange programmes with other member states, research on pharmacy education and practice in the EU, and other matters.

Much of the information for the Czech Republic was provided by the Ministry of Health of the Czech Republic [8].

## 3. Evaluation and Assessment

### 3.1. Organisation of the Activities of Pharmacists, Professional Bodies

Table 2 provides details of the numbers and activities of community pharmacists and pharmacies in the Czech Republic. Items such as competences are expounded upon in the “comments” column.

The data in Table 2 shows that compared with the EU linear regression estimation (for definition and calculation, see Atkinson and Rombaut, reference [1]), the ratio of the actual number of community pharmacists in the Czech Republic (/population) compared with the linear regression estimation for the Czech Republic = 0.84. Thus, the number of pharmacists per population is close to the EU norm. The same comparison for community pharmacies produces a ratio of 0.77.

The activities and occupations of pharmacists in the Czech Republic are similar to those of community pharmacists in other EU member states [1].

Table 3 provides details of the numbers and activities of assistant pharmacists in the Czech Republic.

The legislation of the Czech Republic recognises assistant pharmacists as health care professionals. Although their education and training is in the form of a three-year course, it cannot be compared with a “B. Pharm.”, as defined by the Bologna declaration (see above).

Table 4 provides details of the numbers and activities of hospital pharmacists in the Czech Republic.

The number of hospital pharmacists is low when compared with the EU average. The ratio of the actual number compared with the linear regression estimation = 0.40, (for definition and calculation, see Atkinson and Rombaut, reference [1]). The ratio for hospital pharmacies compared with the EU average is 0.59.

Table 5 provides details of the numbers and activities of industrial pharmacists and pharmacists in other sectors, in the Czech Republic.

Industrial pharmacists in the Czech Republic have similar practices and duties to those in other EU countries [1]. As the numbers of industrial pharmacists were not available for most of the European countries, a comparison with the EU average is not possible.

Table 6 provides information on professional associations for pharmacists in the Czech Republic.

References to the various legislative and other documents [9] concerning pharmacy regulations and practice are found in the appendix.

### 3.2. Pharmacy Faculties, Students, and Courses

Table 7 provides details of pharmacy higher education institutions (HEIs), staff, and students in the Czech Republic.

The education website [11] provides information on the educational system in the Czech Republic as well as study and educational opportunities not only in the Czech Republic but throughout Europe. It also provides links to the legislation regulating education in the Czech Republic (the current wording of the School Act, Higher Education Act, Act on Pedagogical Workers and the White Book, etc.), various documents from the area of education and training; publications from the area of the school system, and selected documents relating to international activities.

It is to be noted that courses are given in Czech and English [12].

Table 8 provides details of the specialisation electives in pharmacy HEIs in the Czech Republic.

Table 9 provides details of past and present changes in pharmacy education and training in the Czech Republic.

### 3.3. Teaching and Learning Methods

Table 10 provides details of student hours by the learning method (for further details on the definitions of the different methods, see Atkinson and Rombaut, reference [1]).

Formal lectures constitute 30% of student hours. Similar percentages are devoted to traineeship and to project work; the latter two take place mainly in the fourth and fifth years of studies. This suggests that student exchange would be easier in these later years, as there would be less need for exact coordination in the timing of deliverance of given subject matters (in lecture, practicals or tutorials) between the host and the home university as in the first three years.

### 3.4. Subject Areas

Table 11 provides details of student hours by subject area (for further details on the definitions of the subject areas, see Atkinson and Rombaut, reference [1]).

Taking the MEDISCI/CHEMSCI ratio as an indicator [13] of the nature of the M. Pharm. degree course (ratio = 658/574 = 1.1), it appears that the course is balanced compared with EU courses that are more “chemical science” e.g., Germany (ratio = 0.7), or more “medicinal science” e.g., Ireland (ratio = 2.6). Generic activities and traineeship make up ((650 + 856)/4300) × 100 = 38% of student hours.

### 3.5. Impact of the Bologna Principles [3]

Table 12 provides details regarding the various ways in which the Bologna declaration impacts on the pharmacy HEIs of the Czech Republic.

Data in the above table are in exchange months per year. They show that there is substantial student exchange, and thus that application of Bologna principles such as ECTS works.

### 3.6. Impact of EU Directive 2013/55/EC [2]

Table 13 provides details regarding the various ways in which the EU directive impacts on pharmacy education and training in the Czech Republic.

The Czech Republic mainly conforms to the different aspects of the EU directive, with notably a five-year tunnel degree. Aspects of the Bologna agreement such as the ECTS and the Diploma Supplement are present.

## 4. Discussion and Conclusions

Regarding the state of pharmacy in the Czech Republic compared with the EU, in many aspects, the activities and occupations of Czech pharmacists are attuned to those in other EU member states, in spite of the fact that the Czech Republic only recently (2004) joined the EU. The numbers of pharmacists in relation to the population are lower than the EU norm. Assistant pharmacists are officially recognised; they undergo a three-year training period. This cannot, however, be assimilated to a bachelor degree in pharmacy.

As in most other EU member states, the profession of hospital pharmacist exists, although this is not recognised by the EU directive. Hospital pharmacists receive their training following graduation, but such training is not organised by the university. Again, as in most EU countries, the Czech pharmacy chamber deals with specific problems relating to the organisation and ethics of practice. The chamber has also an advisory role concerning university courses for pharmacy.

There are two pharmacy HEIs in Hradec Králové and Brno. There is a substantial foreign student intake, especially from Slovakia, and courses are given in Czech and English. Czech pharmacy faculties utilise the ECTS to promote student exchange. These are valid throughout the EHEA. The course is balanced amongst three main elements: lectures, project work, and a pre-graduate traineeship. The course also strikes a balance between medicinal and chemical subjects.

## Figures and Tables

**Table 1:** Health statistics for the Czech Republic [4,5].

Total Population	10, 543, 000
Life expectancy at birth m/f/both sexes (years)	75.9/81.7/78.8
Healthy life expectancy at birth (years)	69.4
Total expenditure on health per capita	2434 $

**Table 2:** Numbers and activities of community pharmacists and pharmacies [9], Appendix A.

Item	Numbers	Comments
Pharmacists	6000	1757 Inhabitants/Pharmacist
Pharmacies	2420 (+251 sub units)	Pharmacists/pharmacy: 2.2 Inhabitants/pharmacy: 3947
Competences and roles of community pharmacists		Supplying prescription and OTC medicines and medical devices, Giving advice on medicines and lifestyle, Compounding of medicines, Keeping records (registration) of narcotic drugs, Ordering of medicines, Services to nursing and care homes, Blood pressure and glycaemia monitoring, Patient counselling service—individual consultations of drug-related problems, Supplying prescriptions for wards in health care facilities, Reporting of adverse drug reactions (ADR) to governmental authorities.
Is ownership of a community pharmacy limited to pharmacists?	No	Any physical or juridical person has legal right to own a public pharmacy [10].
Rules on geographical distribution of pharmacies?	No	There are no governmental restrictions on the geographical distribution of community pharmacies as a function of population density.
Are drugs and health care products available to the general public by channels other than pharmacies?	Yes	The following can deliver some health care products: (i) Veterinary doctors, (ii) Shops selling medical devices, (iii) Medical emergency teams, (iv) Hospitals

**Table 3:** Numbers and activities of assistant pharmacists.

Item	Numbers	Comments
Are persons other than pharmacists involved in community practice?	Yes	In addition to pharmacists, assistant pharmacists are also considered to be professional pharmacy staff.
Their titles and number(s)	4600	In Czech, they are designated as “*Diplomovaný Specialista*” (DiS).
Organisations providing and validating education and training of assistant pharmacists		Education is provided by Medical Colleges and Secondary Medical Schools. Education is validated by passing the final exam, which is called the *Absolutorium*.
Duration of studies (years)	3 years	
Subject areas		English or German, Latin, Information and Communication Technologies, Chemistry and Biochemistry, Psychology and Communication, Health Education, Anatomy and Physiology, Microbiology and Hygiene, Human Nutrition, Pharmaceutical Botany, Analysis of Drugs, Pharmacology, Compounding of Medicines, Laboratory Technology, First Aid, Pathophysiology and Pathology, Pharmacognosy, Pharmaceutical Chemistry, Basics of Radiology, Pharmacy Practice, Public Health Care, Dispensing, Medical Devices, Practical Training.
Competences and roles		Supplying OTC drugs, Medical devices and other health products, Compounding of medicines.

**Table 4:** Numbers and activities of hospital pharmacists.

Item	Numbers	Comments
Does such a function exist?	Yes	The legislation covers: - the area of state-owned hospitals (and hospital pharmacies) - list of pharmaceutical specializations including hospital pharmacy - specialisation curricula, including hospital pharmacy
Number of hospital pharmacists	430	
Number of hospital pharmacies	93	
Competences and roles of hospital pharmacists		Supplying of prescription medicines for wards and outpatient clinics Clinical pharmacy consulting, Compounding of medicines for wards and outpatients, Production of patient-specific medicines (e.g., cytotoxic preparations, all-in-one sterile bags), Supplying of specialised individual medical devices for patients and medical materials for wards, Supplying and check of raw materials for the pharmacy and specialised laboratories of the hospital, Supplying and evidence of narcotic drugs, Adverse effects reporting, Participation in clinical drug evaluation (safety and efficacy), Patient counselling service—individual consultations of drug-related problems, Information service for healthcare professionals.

**Table 5:** Numbers and activities of industrial pharmacists and pharmacists in other sectors.

Item	Numbers	Comments
**Industrial Pharmacy and Pharmacists**		
Number of pharmaceutical companies with production, R&D, and distribution	228	There are 228 licensed distributors in the Czech Republic. There are no reliable sources to divide the producers and distributors according to the mentioned groups.
Companies producing generic drugs only		Zentiva (https://www.zentiva.com/) Teva Pharmaceutical Industries Ltd. (http://www.tevapharm.com/)
Number of pharmacists working in industry	15	These are only the persons registered with the Czech Chamber of Pharmacists. There are possibly many more, but this number is not known, since they need not be registered with the Czech Chamber of Pharmacists.
Competences and roles		Preclinical drug evaluation (safety and efficacy), Clinical drug evaluation (safety and efficacy), Research, Technology, Management, Marketing, Control, Production, Development, Business.
**Pharmacists Working in Other Sectors**		
Number of pharmacists working in other sectors	43	These are only the persons registered with the Czech Chamber of Pharmacists. There are possibly many more, but this number is not known, since they need not be registered with the Czech Chamber of Pharmacists.
Sectors in which pharmacists are employed		Armed forces, Secondary school education and training, Universities, National health services, SUKL (State Institution of Drug Control: registration of drugs—www.sukl.cz), IKEM (Institute of Clinical and Experimental Medicine—clinical trials—www.ikem.cz), Laboratories (research, production, control, development), Distribution, Sales management and marketing.
Competences and roles in other sectors		Education and Training, Research, Management, Control, Production, Consulting, Drug evaluation and registration.

**Table 6:** Professional associations for pharmacists in the Czech Republic.

Item		Comments
Registration of pharmacists	Yes	Registration with the Czech Chamber of Pharmacists (http://www.lekarnici.cz/) is compulsory for all practising pharmacists. The Czech Chamber of Pharmacists is an independent, non-political, autonomous professional organisation responsible for the interests, professionalism, ethics, and honour of the pharmaceutical profession. The law prescribes obligatory membership in the Chamber for all pharmacists practising in pharmacies in the Czech Republic. The Czech Chamber of Pharmacists: Ensures that its members exercise their profession in conformity with the highest professional standards, as well as with the principles of medical ethics and within the law; Serves as the guarantor of professionalism on the part of its members and certifies the fulfilment of the requirements for the practice of medicine; Reviews and defends the rights of the professional; Defends the professional honour of its members; Maintains the register of its members. The Chamber is entitled to: Participate in negotiations concerning the price lists for pharmaceuticals; Take part in competition proceedings to fill leading positions in the health care sector; Establish requirements for practice by its members; Investigate malpractice complaints filed against its members; Issue opinions on the conditions and forms of the Continuing Education of Pharmacists; Participate in specialisation exams. For more information, see the web site: http://www.lekarnici.cz.
Creation of pharmacies and control of territorial distribution	Yes	Territorial distribution of pharmacies is not regulated. Any physical or juridical person has the legal right to open a new pharmacy, but it must receive a licence from the regional District Office.
Ethical and other aspects of professional conduct	Yes	The ethical code of the Czech Chamber of Pharmacists is valid since 2005 (http://www.lekarnici.cz/).
Quality assurance and validation of university courses	Yes	A representative of the Czech Chamber of Pharmacists is a member of the Scientific Council of the Faculty of Pharmacy that approves any changes in the pharmacy curricula.

**Table 7:** Pharmacy higher education institutions (HEIs), staff, and students in the Czech Republic.

Item	Number	Comments			
Number of pharmacy HEIs in the Czech Republic	2	The two HEIs are: Charles University, Faculty of Pharmacy in Hradec Králové (FPCU) (www.faf.cuni.cz) The University of Veterinary and Pharmaceutical Sciences Brno, Faculty of Pharmacy (FPVPU) (http://faf.vfu.cz/).			
Public pharmacy HEIs	2	There are no private pharmacy HEIs in the Czech Republic.			
Faculty attachment		The faculties of pharmacy are independent bodies.			
Do HEIs offer B and M degrees?	No	Only a master degree; a bachelor degree does not exist.			
**Teaching staff**					
Staff (nationals)	190				
Professionals from outside the HEIs	7 (academic)	Staff from Slovakia. There are also 50 staff consisting of: community and hospital pharmacists involved in traineeship, management persons from pharmaceutical industry, psychologists, and economic experts.			
**Students**					
Graduates that become registered pharmacists	200	The data are from the academic year 2014/15. Information about the admission procedure is available at: http://www.faf.cuni.cz/studium/prijimaci_rizeni/bakalarske_magisterske/20112012/Stranky/default/aspx. Twenty-five to 30% of students drop out during the five years of study, and 90% of those who graduate become registered pharmacists (the remaining 10% do not work in pharmacies and need not be registered with the Czech Chamber of Pharmacists).			
Number of places on entry following secondary school	300				
Number of applicants for each entry place	1130	Data from the academic year 2014/15. 4.2 applicants per place.			
Number of EU international students	350	Main origins: 210 from Slovakia, who do not have to learn Czech since the Slovak and Czech languages are very similar; 27 from Greece.			
Number of non-EU international students	26	Kosovo, Kazakhstan, Russian federation, Iran, Iraq, Saudi Arabia, Egypt, Zimbabwe, United Arab Emirates, Vietnam, Belarus, Uzbekistan.			
**Entry requirements following secondary school**					
Specific national entrance examination for pharmacy			Yes		Written tests in biology and chemistry.	
**Fees per year**					
For home and EU students				No tuition fee for courses in Czech. 7600 € for courses in English	
For non EU students				7600 € for courses in English	

**Table 8:** Specialisation electives in pharmacy HEIs.

Item		Comments
Do HEIs Provide Specialised Courses?	No	
Specialisation provided by other organisations?	Postgraduate	Specialisation training for hospital pharmacy Hospital pharmacy specialisation training lasts four years; it includes - four-year practice in pharmacy with at least two years in hospital pharmacy - Several theoretical courses focused on pharmacotherapy, legislation, hospital pharmacy technologies, etc. - Practical training at accredited hospital pharmacies (compounding, sterile preparations, cytotoxic compounding and handling, quality assurance) - Each aspirant must: - pass two tests during training - submit a thesis (within the scope of hospital pharmacy) - pass the board examination to obtain the specialisation diploma in hospital pharmacy.

**Table 9:** Past and present changes in education and training in the Czech Republic pharmacy HEIs.

Item		Comments
Have there been any major changes since 1999?	Yes	Transfer to Bologna credit transfer system, also known as the European Credit Transfer and Accumulation System (ECTS) [3] and the introduction of six months of practical training in the fifth year.
Are any major changes envisaged before 2019?	Yes	As and if required by the directives of the EU.

**Table 10:** Student hours by learning method.

Method	Year 1	Year 2	Year 3	Year 4	Year 5	Total
Lecture	364	350	322	378	0	1502
Tutorial	84	182	157	140	0	560
Practical	280	252	196	98	0	597
Project	0	0	0	168	252	420
Community traineeship	40	0	0	0	960	1000
Industrial/academic traineeship	0	80	0	0	0	80
Electives						
Choice	112	56	84	0	0	252
Optional	0	0	84	64	0	148
**Total**	880	920	840	848	1212	4559

**Table 11:** Student hours by subject area.

Subject Area	Year 1	Year 2	Year 3	Year 4	Year 5	Total
CHEMSCI	168	308	42	56	0	574
PHYSMATH	168	0	14	0	0	182
BIOLSCI	168	98	0	0	0	266
PHARMTECH	0	0	406	336	0	742
MEDISCI	56	280	196	126	0	658
LAWSOC	140	28	112	182	0	462
GENERIC	196	168	28	168	0	560
TRAINEESHIP	0	0	0	0	856	856
Total	896	882	798	868	856	4300

CHEMSOC: chemical sciences; PHYSMATH: physical and mathematical sciences; BIOLSCI: biological sciences; PHARMTECH: pharmaceutical technology; MEDISCI: medicinal sciences; LAWSOC: law and social sciences; GENERIC: generic competences.

**Table 12:** Ways in which the Bologna declaration impacts on the Czech Republic pharmacy HEIs.

Item		Comments
“Comparable degrees with diploma supplement”	Yes	
“Two main cycles (B and M) with entry and exit at B level”	No	There is a five-year “tunnel” degree structure.
“European Credit Transfer System (ECTS) system of credits with links to life-long learning (LLL)”	Yes	The ECTS system of credits was introduced in 2006/2007.
“Addressing obstacles to mobility”	Partial	We offer a parallel pharmacy study programme in English for incoming international students. Incoming Erasmus students receive certain financial support from the Czech Ministry of Education to cover part of their expenses for accommodation. Outgoing Erasmus students receive about 350 € per month financial support from the Czech Ministry of Education.
“Application of European QA”	Yes	The study programmes are regularly accredited by the Accreditation Commission of the Czech Republic, which is a full member of the European Association for Quality Assurance in Higher Education (ENQA) [14].
European dimension		The Faculty of Pharmacy, Charles University, has an agreement on co-supervision of PhD courses with the Faculty of Pharmacy, University of Coimbra, Portugal.
ERASMUS staff exchange to Prague from elsewhere		Staff months: 1 Portugal, Italy, Poland, Lithuania.
ERASMUS staff exchange from Prague to other HEIs		Staff months: 2. Poland, Spain, Italy, Slovenia.
ERASMUS student exchange to Prague from elsewhere		Student months: 140 Portugal, Spain, Poland, Italy, Slovakia, Germany.
ERASMUS student exchange from Prague to other HEIs		Student months: 170 Germany, Sweden, Slovenia, Italy, Portugal, Finland, Great Britain, Austria, Spain, Estonia.

**Table 13:** Ways in which the elements of the EC directive (left column) impact on Czech Republic pharmacy HEIs.

Item	Comments
“Evidence of formal qualifications as a pharmacist shall attest to training of at least five years’ duration…”	The curriculum fulfils the EU requirements.
“…four years of full-time theoretical and practical training at a university or at a higher institute of a level recognised as equivalent, or under the supervision of a university;”	The curriculum fulfils the EU requirements.
“…six-month traineeship in a pharmacy that is open to the public or in a hospital, under the supervision of that hospital's pharmaceutical department.”	We would prefer a compulsory period of four months in community or hospital pharmacy for all students, plus two months either in industry (for those that plan to work in industry after graduation) or an additional two months in a pharmacy for those planning to work in a pharmacy.

## Appendix A. References to Legislation and Other Documents Concerning Pharmacy Organisation, Regulation and Practice

- Czech Pharmacopoeia 2009 and previous including the Supplements—GRADA Publishing
- SUKL (State Institution of Drug Control) Official Journals and Regulations—www.sukl.cz
- Czech Republic Statutes at Large
- Czech republic Ministry of Health Official Journals and Directives
- Health Insurance Institutions rules—www.vzp.cz for example
- Constitutional Code No. 1/1993
- Code No. 40/1964, Civil Code
- Code No. 513/1991, Business Law
- Code No. 65/1965, Labour Code
- Code No. 140/1961, Punity Law
- Code No. 378/2007, Law on Drugs
- Council Directive 89/105/EEC, of 21 December 1988, relating to the transparency of measures regulating the pricing of medicinal products for human use and their inclusion within the scope of national health insurance systems.

Bibliographic references:

- Český lékopis, Praha, Grada Publishing, actual edition
- Journals—Časopis českých lékárníků, Praktické lékárenství, Zdravotnické noviny
- Smečka V., Rusek V., Kolář J.: Lékárenství I., 1. vyd., VFU, Brno 2008
- Kolář J., Smečka V.: Lékárenství II, 1.vyd., VFU, Brno, 2008
- Solutio-příruční kniha pro lékárny, Praha, Medon 1996–2004
- Lenka Práznovcová, Ladislav Strnad: Farmakoekonomika pro lékaře, farmaceuty a manažery zdravotnických zařízení, Maxdorf, ISBN80-73450488.
- Lenka Práznovcová, Ladislav Strnad: Zdraví, zdravotnictví a léková politika v ČR a v zemích EU, Nakladatelství Maxdorf, ISBN 80-807345-075-5.
